# 

**Published:** 1947

**Authors:** 


					Vol. LXIV. No. 232.
,yx
WINTER, 1947.
The Bristol
Medico-Chirurgical Journal
PUBLISHED UNDER THE AUSPICES OF
THE BRISTOL MEDICO-CHIRURGICAL SOCIETY.
Editor: J. A. NIXON, C.M.G., M.D., F.R.C.P.
WITH WHOM ABE ASSOCIATED
A. RENDLE SHORT, M.D., b!s., B.Sc., F.R.C.S.
W. MELVILLE CAPPER, M.B., B.S., F.R.C.S.
G. E. F. SUTTON, M.C., M.D., B.S., M.R.C.P.
y *, " \
E. WATSON-WILLIAMS,. M.p., ChiM.; F.R.C.S., Editorial Secretary.
/ /
MB "? . 8' I ? ?
$8 '- fi. " * ? ?'? ?
,
BRISTOL:
J- W. ARROWSMITH LTD., QUAY STREET & SMALL STREET
Price Four Shillings net.
j

				

